# Off-label use of cancer therapies in women diagnosed with breast cancer in the United States

**DOI:** 10.1186/s40064-015-0981-z

**Published:** 2015-05-01

**Authors:** Sophie Hamel, Douglas S McNair, Nicholas J Birkett, Donald R Mattison, Anthony Krantis, Daniel Krewski

**Affiliations:** McLaughlin Centre for Population Health Risk Assessment, University of Ottawa, Ottawa, ON Canada; Department of Cellular and Molecular Medicine, University of Ottawa, Ottawa, ON Canada; Cerner Corporation, Kansas City, Missouri USA; School of Epidemiology, Public Health and Preventive Medicine, University of Ottawa, Ottawa, ON Canada; Risk Sciences International, Ottawa, ON Canada

**Keywords:** Off-label use, Oncology, Breast cancer, Pharmacoepidemiology, Prescribing practices, Clinical pharmacology, Women’s health

## Abstract

**Purpose:**

To determine the level of off-label cancer therapy use in a population of female breast cancer patients and to establish whether this use was evidence-based.

**Methods:**

A study was conducted by sampling Cerner’s data warehouse for all women diagnosed with breast cancer between January 2000 and June 2009 who received at least one cancer therapy approved by the US-FDA during the study period. Drug encounters were considered off-label if the circumstances of use did not match the age or medical diagnoses specified on the product label at the time of study. The level of evidence for the use of these drugs in a breast cancer setting was evaluated from randomized phase III trials using a tiered approach.

**Results:**

The study included 2,663 women with a median age of 59 years. A total of 1,636 off-label encounters were recorded, representing 13.0% of all encounters. Of the 65 cancer therapies investigated, 55.4% were prescribed off-label. The drugs with the highest off-label use were, in a descending order, vinorelbine, carboplatin, bevacizumab, leuprolide, liposomal doxorubicin and cisplatin. Most off-label encounters were evidence-based and more likely to be associated with private insurance coverage, younger age, ethnicities other than Caucasian, smaller treatment centres and drugs with limited labeled indications that have a longer market history.

**Conclusions:**

Off-label prescribing is common practice in oncology and is an integral component of breast cancer treatment strategies. While this practice tends to be associated with specific socio-demographic factors and disease characteristics, the majority of off-label encounters appear to be evidence-based.

## Background

All prescription drugs are labeled in accordance with the circumstances of use and evidence collected from randomized controlled clinical trials. However, once a drug reaches the market, a physician may exert clinical judgement and prescribe drugs for other conditions or circumstances. This type of prescribing is considered 'off-label’ and has become part of mainstream medical practice extending beyond the specifications of the drug label (American Society of Clinical Oncology 2006; Poole & Dooley 2004; United States General Accounting Office 1991).

Off-label use in breast cancer has been previously reported (United States General Accounting Office 1991; Delpeuch et al. 2011), but only one study has focused on the off-label chemotherapeutic use in women over 65 years old diagnosed with breast cancer between 1991 and 2002 (Dean-Colomb et al. 2009). Although off-label use of chemotherapy has been reported, breast cancer patients are treated with a wide variety of agents which are not considered typical chemotherapies. The discovery of distinct molecular determinants of tumor development and progression has opened a new era of targeted therapies. Investigating the extent of off-label use, taking into consideration the broader range of therapies available and extending the analysis to the overall breast cancer population, regardless of age, may provide insight into the scope of this practice.

Consequently, we sought to estimate the extent of off-label use of any chemotherapies, targeted agents, hormone therapies, and immunotherapies approved by the FDA as a cancer therapy at the time of this study and investigate whether drug coverage or factors such as patient demographics, drug, treatment centre and physician characteristics influence off-label prescribing among breast cancer patients.

## Results

### Number of drugs used beyond the label specifications

Among the 107 drugs considered in this study, 43 chemotherapies and 22 other cancer therapies were administered to a population of 2,633 breast cancer patients totaling 14,586 drug encounters. Only 21 (32.3%) of these therapies were indicated for breast cancer during the full study period or at the time of their first introduction to the market. Gemcitabine was approved by the FDA for breast cancer in December 2004. Accordingly, all gemcitabine encounters which occurred before that date were considered off-label if no indication listed in Table 1 was diagnosed. It is of note that, although the FDA revoked breast cancer as an indication for bevacizumab on November 18, 2011 (FDA News Release 2011), breast cancer was considered a labeled indication from the original approval date for this indication (February 22, 2008) until the end of the study period. A total of 36 drugs (55.4% of all cancer therapies administered) were used either for an off-label indication or by a recipient whose age differs from the label specifications. A total of 13.0% of patients received at least one cancer therapy under these off-label circumstances.

**Table 1:** **Summary of FDA-approved chemotherapeutic agents investigated in current study by category and subcategory**

**Drug category**	**Drug name**	**Indicated for breast cancer**	**Labeled indications if not indicated for breast cancer (ICD-9 codes)**	**Initial FDA approval during study period**
Alkylating agents	Carboplatin		183, 198.6	1989
	Carmustine		191,200-202,203.0	1977
	Chlorambucil		200-202,204.1,204.9	1957
	Cisplatin		183,186,188,198.6	1978
	Cyclophosphamide	Yes		1959
	Dacarbazine		172,198.2, 201	1975
	Ifosfamide		186	1988
	Mechlorethamine		162.2-162.5,162.8,162.9,197.0,200,201,202.0	1949
			202.1,202.8,204.1,205.1,208.1,238.4,511.9	
	Melphalan		183,^1^ 198.6, ^1^203.0	1964
	Oxaliplatin		153,154,197.5	August 2002
	Streptozocin		157.4	1982
	Thiotepa	Yes		1959
Antimetabolites	Azacitidine		205,280-285	May 2004
	Capecitabine	Yes		1998
	Cladribine		202.4	1993
	Clofarabine		204.0,204.9	December 2004
	Cytarabine		204.0, 204.9, 205.0, 205.1, 205.9	1969
	Fludarabine		204.1, 204.9	1991
	Fluorouracil	Yes		1962
	Gemcitabine	Yes^2^	157.0-157.9, 162.2-162.5, 162.8, 162.9, 197.0^3^	1996
	Hydroxyurea		172.1-172.4, 173.1-173.4, 183, 195.0, 198.2, 198.6, 205.1, 205.9	1967
	Mercaptopurine		204.0, 204.9	1953
	Methotrexate	Yes		1953
	Pemetrexed		162.2-162.5, 162.8, 162.9, 163, 197.0	February 2004
Anti-tumor antibiotics	Bleomycin		140-149, 160, 161, 180, 184, 186, 187, 195.0, 197.2, 198.82, 200–202, 511	1973
	Dactinomycine	Yes^4^		1964
	Daunorubicin		204.00-204.91, 205.00-205.21, 205.80-205.91, 208.00-208.01	1979
	Doxorubicin	Yes		1974
	Epirubicin	Yes		1974
	Idarubicin	Yes		1999
	Liposomal doxorubicin		205.0, 205.9	1990
	Mitomycin-C		151, 157	1981
Topoisomerase inhibitors	Etoposide		162.2-162.5, 162.8, 162.9, 186, 197.0	1983
	Irinotecan		153, 154, 197.5	1996
	Mitoxantrone		185, 205–208, 340	1987
	Topotecan		162.2-162.5, 162.8, 162.9, 180, 183,197.0, 198.6	1996
Anti-mitotic	Docetaxel	Yes		1996
	Ixabepilone	Yes		October 2007
	nab-Paclitaxel	Yes		January 2005
	Paclitaxel	Yes		1992
	Vinblastine	Yes		1965
	Vincristine		171, 189.0, 189.9, 194.0, 194.9, 198.0, 200, 201, 202.0, 202.1, 202.8, 204.0, 204.9	1963
	Vinorelbine		162.3-162.5, 162.8, 162.9	1994

^1^Indication for the oral formulation.

^2^FDA-approved for breast cancer on December 18, 2004.

^3^Indications approved by the FDA before December 18, 2004. Ovarian cancer only included after it was approved by the FDA on July 14 2006.

^4^Not breast cancer specifically but locoregional solid tumors.

### Off-label use by drug category

The frequency of off-label use of chemotherapy and other drugs was similar (55.8% and 54.5%). However, off-label patient encounters was almost double for chemotherapy compared to other drugs (14.1% vs 8.4%) (Table 2). Topoisomerase inhibitors and alkylating agents were the classes of chemotherapies with the highest proportion of off-label use, whereas the targeted agents ranked first for the other cancer therapies.

**Table 2:** **Number of off-label drugs and encounters for each drug category and subcategory**

**Category**	**Number off-label drugs (%)** ^**1**^	**Off-label encounters (%)** ^**2**^	**Subcategories**	**Number Off-label drugs (%)** ^**2**^	**Off-label encounters (%)** ^**2**^
**Chemotherapies**	24 (55.8%)	1153 (14.1%)	Topoisomerase inhibitor	4 (100%)	61 (27.9%)
			Alkylating agents	7 (58.3%)	485 (26.2%)
			Anti-metabolites	8 (61.5%)	103 (8.5%)
			Anti-tumor antibiotics	3 (37.5%)	75 (6.3%)
			Anti mitotics	2 (28.6%)	429 (11.6%)
**Other agents**	12 (54.5%)	483 (8.4%)	Targeted therapies	2 (50.0%)	11 (37.9%)
			Immunotherapies	5 (45.5%)	233 (8.2%)
			Hormone therapies	5 (62.5%)	239 (8.2%)

^1^Expressed as a percentage of all drugs including those indicated for breast cancer in a given therapeutic category or subcategory.

^2^Expressed as a percentage of all encounters including those indicated for breast cancer in a given therapeutic category or subcategory.

### Age-related off-label use

Only 8 drugs which we considered had age-related restrictions applied by the FDA when they granted approval (either limiting approval based on age or to a particular age-related event such as menopause) (Table 3). Most encounters with these drugs (96.4%) were in line with the age-related label specifications. Although clorafarabine is indicated for use in pediatric patients with acute or relapse lymphoblastic leukemia, all reported encounters involved patients over the age of 21 diagnosed with other off-label indications than breast cancer.

**Table 3:** **Age as per FDA-approved product label for drugs under study with such specification**

**Drug name**	**Labeled age (years)** ^**1**^
Clofarabine	1-21
Fluoxymesterone	≤50
Goserelin	≤50
Anastrozole	≥50
Exemestane	≥50
Fulvestrant	≥50
Letrozole	≥50
Gemtuzumab ozogamicin	≥50

^1^For the purpose of this study, age of menopause was fixed at 50 years.

### Level of evidence

If we only consider therapies not specifically indicated for the treatment of breast cancer, 55.5% of drug encounters recorded in the database can be categorised as off-label on the basis of indication for use. The drugs with the highest number of off-label uses were, in descending order, vinorelbine, carboplatin, bevacizumab, leuprolide, liposomal doxorubicin, and cisplatin (Table 4). Overall, 93.3% of these off-label drug encounters were associated with a diagnosis of breast cancer. The pattern of off label-use appeared to be aligned with the available evidence to support the use. The level of evidence derived from the drug with the highest off-label use, vinorelbine, was considered to be sufficient to support its use in a breast cancer setting. During the study period, the other five drugs with the highest off-label use were considered to have limited evidence to support their use in a breast cancer population, including bevacizumab which obtained market approval for this indication in February 2008 and was subsequently revoked by the FDA in November 2011 after concluding that the drug has not been shown to be effective or safe for that use.

**Table 4:** **Level of evidence for the 6 drugs with the highest off-label use**

**Drugs**	**Total**	**Total off-label (%)**	**BC specific off-label**	**Level of evidence**	**Primary ICD9 diagnosis code other than BC**
Vinorelbine	732	413 (27.0%)	395	Sufficient	147, 184.4, 188, 202.8, 204.1
Carboplatin	444	382 (25.0%)	338	Limited	147, 162, 182, 191, 202.88, 235.7
Bevacizumab	132	199 (13.0%)	222	Limited	162.9, 184.4, 191.92, 202.8
Leuprolide	136	133 (8.7%)	122	Limited	171, 173.3
Liposomal Doxorubicin	104	67 (4.4%)	59	Limited	162.8, 171.9, 202.8
Cisplatin	68	48 (3.1%)	23	Limited	149.8, 150, 151, 162, 170.3, 173.3, 182, 195.0, 201, 202.8, 203,

The total number of off-label encounters and percentage of total encounters is listed for each drug. The level of evidence and recommendation for the off-label use associated to a breast cancer diagnosis were graded as Sufficient, Limited or Inadequate based on the source and strength of the evidence available during the study period. If the off-label encounter was associated to another condition, the ICD-9-CM code was recorded.

^1^All bevacizumab encounters before February 22, 2008.

^2^Glioblastoma indication approved by FDA (May 05, 2009) after the drug was administered BC = breast cancer.

### Patient demographics

The median age of the study population was 59 years. Drug encounters were more likely to be off-label among younger women (<50 years age) (16.2%), whereas encounters for patients in the older age group (≥75 years) were less common (7.4%). There were more off-label encounters among African American breast cancer patients and patients with other ethnic backgrounds than among Caucasian patients (Table 5). There was no apparent relationship between marital status and the off-label administration of cancer therapies.

**Table 5:** **Demographic, treatment centre, insurance coverage and drug characteristics comparison between on-label and off-label encounters**

**Variable**	**Variable category**	**Off-label (%)**
Age*	15-49	621 (16.2%)
	50-64	396 (10.4%)
	65-74	432 (9.7%)
	75+	187 (7.4%)
Ethnicity*	Caucasian	1113 (10.3%)
	African American	185 (13.0%)
	Other	95 (15.3%)
	Unknown	243 (14.4%)
Marital status	Married	338 (11.0%)
	Other	1293 (11.3%)
	Unknown	5 (10.4%)
Prescription drug coverage^*^	Medicare	209 (9.5%)
	Medicaid	81 (10.4%)
	HMO/PPO	24 (6.9%)
	Blue Cross	65 (15.3%)
	Self-pay	21 (11.0%)
	Other	75 (12.3%)
	Unknown	1161 (11.6%)
Census region^*^	Midwest	255 (8.9%)
	South	264 (11.4%)
	Northeast	1088 (12.0%)
	West	29 (9.0%)
Bed size^*^	1-99	477 (16.2%)
	100-199	48 (5.7%)
	200-299	315 (10.0%)
	300-499	234 (9.4%)
	500+	562 (10.9%)
Physician type^*^	Specialists	751 (10.9%)
	Surgeons	22 (5.9%)
	Generalists	28 (6.3%)
	Unknown	835 (12.1%)
Number of approved indications^*^	1-2	1395 (27.6%)
	3-4	206 (3.1%)
	5-7	34 (9.5%)
	8+	1 (0.0%)
Date of FDA approval^*^	Before 1981	130 (3.2%)
	1981-1990	602 (78.7%)
	1991-2000	635 (7.8%)
	2001-2009	269 (16.1%)

*Differences amongst categories for this variable are significantly different (p < 0.00001) based on a chi-square test of homogeneity.

Expressed as the number and percentage of off-label encounters.

Expressed as the number and percentage of off-label encounters.

### Drug characteristics and insurance coverage

Off-label use was inversely related to the number of labeled indications specific to the drugs under study. Drugs with only one or two labeled indications were more likely to be involved in an off-label drug encounter. The date of market approval was also predictive of off-label use, with therapies approved by the FDA between 1981 and 1990 being more likely to be with linked to off-label use. The information about drug coverage plans was missing for a large proportion of encounters (77.4%); however, considering only encounters for which insurance information is available, Blue Cross was more strongly associated with off-label use.

### Treatment centre and physician characteristics

Although 114 treatment centres were contributing to the database at the time of the study, 67 distinct healthcare institutions provided electronic records of drug encounters meeting the criteria of the study. Of these institutions, most administered cancer therapies in accordance with the label specifications (66 centres), while 47 centres dispensed at least one therapy under off-label circumstances. This practice was more common in treatment centres located in the Northeastern region of the United States, whereas institutions in the Midwest were less likely report this type of use. Smaller centres with a limited number of beds were more likely to administer drugs in an off-label fashion; this practice was less likely in medium size institutions (199–399 beds). Specialists were more inclined to prescribe chemotherapies and other cancer therapies, but none of the physician types appeared to be associated with increased off-label use.

## Discussion

In the present study, 13.0% of women with breast cancer were prescribed at least one cancer therapy under off-label circumstances, less than the 35% reported previously (Dean-Colomb et al. 2009). This difference may be related to the greater diversity of patients and types of therapies considered in our investigation, as well as the disparity between the two study periods. Several drugs were approved by the FDA between 2002 and 2009, including 4 drugs with a labeled breast cancer indication (bevacizumab, gemcitabine, ixabepilone and nab-paclitaxel). The introduction of new medications on the market and the approval of new indications for established drugs may also explain the smaller proportion of women who experienced off-label use in our study, especially since gemcitabine was listed as the second drug with the most off-label use in the previous investigation. In agreement with previous research, vinorelbine demonstrated the highest proportion of off-label use. Off-label and subsequently labeled encounters with bevacizumab was sustained throughout this study. It would be interesting to determine if the use of this drug for treatment of breast cancer has decreased since the FDA revoked this indication from the product label on November 18, 2011.

A previous report showed appreciable geographic variation in prescribing patterns in the US (Wennberg 2004). In the present paper, about two thirds of encounters were recorded in treatment centres located in the Northeastern census region, which includes 46 of the 114 treatment centres in the HealthFact^TM^ database. Since this region represented 19% of the US population in the 2000 census (United States Census Bureau 2010), the data may not be fully representative of overall off-label prescribing practice in the US.

Although specialists were more likely to prescribe cancer therapies, no notable differences were observed in off-label prescribing habits by physician type, an observation which differs from a previous report which focused on a pediatric population, a vulnerable subgroup rarely included in clinical trials (Schrim et al. 2003). Accordingly, the scarcity of evidence to support pediatric off-label use for certain indications may partially explain why specialists, who are most familiar with the patient’s condition, were more inclined to prescribe off-label. Off-label therapy may also be a last resort for patients exhibiting poor prognoses and limited therapeutic options, regardless of physician type.

Off-label encounters were more common in younger compared to older patients. There was no apparent relationship between off-label encounters and marital status, although off-label encounters were more prevalent among non-Caucasians. While other studies have shown racial difference in breast cancer treatment decisions (Griggs et al. 2007; Maly et al. 2006), the literature is ambivalent regarding the association between race and off-label prescribing (Schrim et al. 2003; Maly et al. 2006; Patkar et al. 2007).

As in previous research, we observed that medications with the fewest labeled indications had the highest rate of off-label use (Radley et al. 2006). Therapies approved in the 1980’s were more likely to be used off-label, possibly because older drugs will have had greater opportunity for discovery of new evidence to support off-label use (Demonaco et al. 2006; Eguale et al. 2012). Since these drugs no longer enjoy patent protection, it is unlikely that a randomized clinical trial to provide evidence to support a new indication will be undertaken. Of the 12 new drugs approved by the FDA for the treatment of cancer between 1981 and 1990, only one was indicated for breast cancer; this may explain the high off-label use observed (78.7%) for drugs originally approved during this decade. Drugs marketed before 1981 were less likely to be prescribed off-label, even though physicians may be more familiar with these drugs; these drugs tended to have a higher number of labeled indications, thereby decreasing the likelihood of being prescribed off-label. Private insurers such as Blue Cross demonstrated greater off-label use than public insurers, as reported previously (Patkar et al. 2007), possibly due to government policies restricting access to therapies. Although not evaluated in this article in absence of information on efficacy, the likelihood of off-label use may also depend on the potential therapeutic effect of the drug.

Study strengths include (1) the availability of data from different insurers (2) inclusion of all, not just post-menopausal, breast cancer patients (3) consideration of age in determining off-label use, and (4) the investigation of all approved cancer therapies, not just chemotherapies.

Our study also has a number of limitations. (1) Because ICD-9-CM codes were used in identifying off-label use, any underreporting or miscoding could affect the accuracy of the results. (2) Missing data could influence the results. For example, information on insurance coverage was unavailable for more than 70% of drug encounters. For the purpose of this study, it was assumed that the distribution of the missing information was proportional to that observed within the available data. (3) Since treatment centres in the Northeast region were overrepresented, results may not be representative of overall off-label practice in the United States. (4) Since the data were collected from hospital encounters, this analysis only considers prescriptions filled within the hospital setting; encounters with medications dispensed outside of this setting were not captured. Accordingly, encounters with oral medications such as endocrine therapies may be underrepresented as these drugs are often administered daily over a period of several years. (5) The chi-square tests used evaluate the significance of differences in off-label use with respect to the demographic, treatment centre, insurance coverage, and drug characteristics in Table 6 do not take into account the correlation among repeated encounters with the same patient. However, it is unlikely that even a high degree of correlation among repeat encounters would render these significant differences non-significant (P > 0.05). (6) The association between different disease characteristics and off-label use was not assessed since information on tumor stage, grade, and tumor markers was unavailable. (7) The definition of off-label was conservative, since concomitant or sequential medication (first, second or later line), dosage, frequency, route of administration, and duration of treatment were not considered in this analysis.

**Table 6:** **Summary of other FDA-approved anticancer therapies investigated in current study by category and subcategory**

**Drug category**	**Drug name**	**Indicated for breast cancer**	**Labeled indications if not indicated for breast cancer (ICD-9-CM codes)**	**Initial FDA approval during study period**
Hormone therapies	Anastrozole	Yes		1995
	Exemestane	Yes		1999
	Fluoxymesterone	Yes		1956
	Fulvestrant	Yes		April 2002
	Goserelin	Yes		1989
	Letrozole	Yes		1997
	Leuprolide	No	185	1985
	Tamoxifen	Yes		1977
Targeted therapies	Bortezomib	No		May 2003
	Erlotinib	No		November 2004
	Imatinib	No	150-154, 158.0, 159.0, 159.8, 159.9, 171.5-171.9, 205.1, 205.9, 238.1, 239	May 2001
	Sorafenib	No	155.0, 155.2, 197.7, 189.0, 189.8, 189.9, 198.0	December 2005
Immunotherapies	Aldesleukin	No	172, 189.0, 189.1, 189.8, 189.9, 198.0, 198.2	1992
	Alemtuzumab	No	204.1, 204.9	May 2001
	Bevacizumab	Yes^1^	153, 154, 162.2-162.5, 162.8, 162.9, 191, 197.0, 197.5, 198.3	February 2004^2^
	Cetuximab	No	140-149, 153, 154, 160, 161, 173.0-173.4, 195.0, 197.5	February 2004
	Denileukin diftitox	No	202.1, 202.2	1999
	Gemtuzumab ozogamicin	No	205.0, 205.9	2000
	Panitumumab	No	153, 154, 197.5	September 2006
	Rituximab^3^	No	200, 202, 204.1, 204.9, 714	1997
	Thalidomide	No	0.17.1, 695.2, 203.0	1998
	Trastuzumab	Yes		1998

^1^Breast cancer indication approved by FDA on February 22, 2008 and revoked in November 2011.

^2^Colorectal cancer indication approved in February 2004, Non-small cell lung carcinoma in October 2006, Glioblastoma in May 2009.

^3^Obtained FDA approval for Wegener’s Granulomatosis (WG) and Microscopic Polyngiitis (MPA) only in April 2011 (post-study period).

The specificity of this study to off-label practice in the US between January 2000 and June 2009 should also be noted. Off-label prescribing practices may vary from one jurisdiction to another: although vinorelbine is approved for use in the treatment of breast cancer in most European countries, this indication has not yet been approved in the US.

## Conclusions

Our study corroborates and expands on previous findings to suggest that off-label use of cancer therapies is widespread among patients with breast cancer and that the majority of these encounters have some evidence to support their use. Socio-demographic, insurance coverage, treatment center, and physician and drug characteristics appeared to influence off-label prescribing patterns. More research is warranted to determine whether the practice of off-label prescribing in the context of cancer treatment yields substantial clinical benefits. In the interim, decisions to prescribe a therapy under off-label circumstances should be evidence-based in an effort to achieve therapeutic benefit and to minimize the risk of possible adverse reactions associated with these therapies.

## Methods

### Data source

All female patients from the Cerner HealthFacts^TM^ data warehouse diagnosed with breast cancer (ICD-9-CM 174, 238.3, 239.3) from January 2000 to June 2009 who received at least one cancer therapy in a hospital setting during this period were considered for inclusion in this study. At the time of the analysis, HealthFacts^TM^ contained information on distinct inpatient admissions, emergency department, and outpatient visits from 114 hospitals and clinics throughout the United States.

### Study eligibility criteria

For each patient, the date of the earliest breast cancer diagnosis was designated as the index date. Those who had less than 6 months recorded care prior to the index date were excluded because they were considered less likely to have their breast cancer therapies and potential comorbidities completely recorded. The study was limited to those at least 20 years of age as of their index date.

### Identification of anticancer therapy under study

Any medication approved by the FDA during the follow-up the period for its anticancer properties was considered for inclusion and was classified as chemotherapy, targeted therapy, hormone therapy, or immunotherapy based on its mechanism of action as per Table 7. The chronological FDA-approval history for each drug in a given therapeutic class was validated through the FDA’s Drug Approvals and Databases. To be considered, the drug and its labeled indications had to be endorsed by the FDA at the time of the study. Drugs and indications that were approved during the study period were also included.

**Table 7:** **Categories and subcategories of cancer drugs considered for analysis**

**Category**	**Subcategory**
Chemotherapies	Alkylating agents, Antimetabolites, Anti-tumor antibiotics, Topoisomerase inhibitors, Anti-mitotic agents
Hormone therapies	Estrogen receptor modulators, Aromatase inhibitors, anti-Androgens
Targeted thrapies	Small-molecule inhibitors, Differentiating agents
Immunootherapies	Monoclonal antibodies, Non-specific Immunotherapies andadjuvants, Immunomodulators

All therapies in these categories and subcategories that were FDA-approved for a cancer indication during the study period were evaluated.

### Determination of off-label status

For the purpose of this study, we ascribed off-label status to all drug uses which met at least one of the following criteria: (1) none of the ICD-9-CM codes in the patient’s electronic record during the study period could be matched to any labeled indications of the prescribed drug (Table 1 and Table 6), or (2) the age of the recipient differs from the label specifications (Table 3). An attained age of 50 years was used as an indicator of post-menopausal status as described elsewhere (Lenz et al. 2002; La Vecchia et al. 1997; Kreiger et al. 1992). Off-label uses related to sequential or concomitant therapies (first, second or later line), dosage, duration of treatment, or route of administration were not considered. The main source for determining labeled indications were the drug labels at the time of use found on the FDA website.

For each drug encounter, all associated ICD-9-CM codes, regardless of their diagnostic priority, were considered to determine the indication for use. If none of the ICD-9-CM codes specific to the drug encounter were labeled indications, the overall ICD-9-CM diagnosis information for the patient was investigated. If the patient had never been diagnosed with a condition approved by the FDA for the specific drug, the use was considered off-label. Encounters with drugs that have obtained FDA approvals for new indications during the study period were considered off-label before the official approval date. For labeled indications that could not be accurately coded using the ICD-9-CM system, all codes related to the condition were adopted. For example, since no official ICD-9-CM diagnosis codes exist to specify cancer subtypes expressing a particular tumor marker, the ICD-9-CM codes for breast cancer (ICD-9-CM 174, 238.3, 239.3) were considered labeled indications for therapies indicated for breast cancer tumors overexpressing Her-2. Drugs considered and approved by the FDA at the time of study but not prescribed to the studied patient population are listed in Table 8.

**Table 8:** **Other FDA-approved anti-cancer therapies at the time of study but not used in the studied population**

**Category**	**Drug name**
Chemotherapies	Altretamine, Asparaginase, Bendamustine, Busulfan, Decitabine, Estramustine, Floxuridine, Liposomal Cytarabine, Lomustine, Nelarabine, Pegaspargase, Pentostatin, Procarbazine, Temozolomide, Teniposide, Thioguanine, Valrubicin
Hormone therapies	Bicalutamide, Degarelix, Flutamide, Histrelin, Nilutamide, Toremifene, Triptorelin
Targeted therapies	Dasatinib, Everolimus, Lapatinib, Nilotinib, Sorafenib, Temsirolimus, Vironostat. Bexarotene, Tretinoin
Immunotherapies	BCG, Ibritumomab, Imiquimod, Interferon alfa-2a, Lenalidomide, Tositumomab

### Level of evidence

The six drugs with the highest off-label use were subjected to a more detailed investigation. The off-label uses were categorised as breast cancer specific if (1) a breast cancer diagnosis was associated with the drug encounter, and (2) no other malignancies or known labeled conditions were diagnosed as part of the same encounter (excluding secondary malignant neoplasms defined by ICD-9-CM 196–198). An extensive search of Medline, U.S. FDA clinical reviews, and drug/disease-state databases (UptoDate online, MICROMEDEX) was conducted to further characterize the level of evidence for the off-label uses. All six drugs investigated had evidence derived from at least one well-designed randomized, phase III clinical trial (RCT) in a breast cancer population. All applicable published evidence derived from these RCTs, including those with negative or equivocal findings, were used to rate the overall evidence available for each off-label drug-indication pair. Categorization of available evidence was conducted independently by two of the authors (SH and DM) as well as an independent reviewer using the following three tiered approach: 1) 'sufficient” evidence was derived when at least five RCTs reported reasonable evidence of therapeutic benefit in breast cancer setting; 2) 'limited" evidence was extracted from at least one RCT but the conclusions from overall findings are inconsistent; and 3) 'inadequate’ evidence was derived when no RCT reported benefit or non-inferiority when compared to the standard chemotherapy regimen.

### Statistical analysis

The level of off-label use was calculated by dividing the number of off-label drug encounters by the total number of encounters for a given drug and therapeutic class. Additionally, the number of drugs used under off-label circumstances was calculated using all drugs within a given therapeutic category or subcategory as a denominator. Variation in the percentage of off-label encounters with patient age, ethnicity, marital status, source of payment, census region, bed size range, physician’s specialty, the date of market approval for a drug, and the number of approved indications was examined using chi-square tests. All analyses were conducted using SAS software (Version 9.2, SAS Institute, Cary, NC).
